# Co-Formulation of Iron Oxide and PLGA Nanoparticles to Deliver Curcumin and IFNα for Synergistic Anticancer Activity in A375 Melanoma Skin Cancer Cells

**DOI:** 10.3390/pharmaceutics17070860

**Published:** 2025-06-30

**Authors:** Magdi Abobaker, Mershen Govender, Yahya E. Choonara

**Affiliations:** 1 Wits Advanced Drug Delivery Platform Research Unit, Department of Pharmacy and Pharmacology, School of Therapeutic Sciences, Faculty of Health Sciences, University of the Witwatersrand, 7 York Road, Parktown, Johannesburg 2193, South Africa; 2 Wits Infectious Diseases and Oncology Research Institute, Faculty of Health Sciences, University of the Witwatersrand, Johannesburg 2193, South Africa

**Keywords:** melanoma, synergistic treatment, controlled release, chemotherapy, immunotherapy, curcumin, interferon alpha, A375 melanoma cells

## Abstract

**Background/Objectives**: Skin cancer remains a significant global health issue, driving the development of new treatment strategies to improve clinical outcomes and prevent recurrence. Traditional monotherapies often face obstacles such as bioactive resistance, prompting interest in combination therapies that enhance efficacy, while minimizing side effects. This study investigated the use of a co-nanoparticle approach of iron oxide nanoparticles (NPs) surface-functionalized with curcumin (Cur-FeONPs) delivered with prolonged-release interferon alpha (IFNα)-loaded PLGA NPs (IFNα-PLGANPs) for the synergistic treatment of malignant melanoma tested in A375 cells. **Methods**: Extensive in vitro characterization studies of the Cur-FeONPs and IFNα-PLGANPs were performed, including zeta-size profiling, morphological studies, and structural validation, in addition to cytotoxicity assessments on A375 melanoma and NIH-3T3 fibroblast cells. **Results**: The Cur-FeONP and IFNα-PLGANPs synthesis processes yielded NPs with an average size of 111.0 nm and 97.0 nm, respectively. Morphological and structural validation studies determined the successful synthesis of the nanoparticulate systems, with cell viability analyses displaying significant cytotoxicity against A375 melanoma cells for the combination treatment, when compared to the individual platforms, with a minimal effect on NIH-3T3 fibroblast cells. **Conclusions**: The results of this study present a promising synergistic approach for enhanced anticancer activity in A375 melanoma skin cancer cells, providing a potential platform for future preclinical and clinical studies.

## 1. Introduction

Skin cancer is one of the most prevalent malignancies worldwide, necessitating diverse treatment strategies to enhance therapeutic outcomes and prevent recurrence. It primarily manifests as non-melanoma skin cancer (NMSC) and malignant melanoma (MM), with NMSC being more common, albeit with a decreased mortality [1,2]. Treating MM ≥ 1 mm in size typically involves surgical resection followed by radiotherapy or chemotherapy. However, conventional therapies are often hindered by drug resistance, suboptimal efficacy, and significant side effects due to nonspecific targeting, which can damage healthy tissues [3,4].

To overcome the shortfalls of such monotherapies, later anticancer interventions use combination therapy (i.e., multiple therapeutic agents) to enhance chemo-drug efficacy. For example, radiotherapy is combined with chemotherapy or immunotherapy using checkpoint blockade to address both NMSC and MM [5].

Nanoparticle (NP)-based therapies have emerged as promising alternatives to conventional treatments due to their ability to enhance drug delivery and minimize systemic toxicity. Among them, iron oxide nanoparticles (FeONPs) have gained significant attention in anticancer research due to their ease of synthesis, low toxicity, and multifunctional applications, including ferrofluids, hyperthermia treatment, magnetic resonance imaging (MRI), and targeted drug delivery [6,7]. The versatility of FeONPs further allows it to be surface-modified with a range of bioactives for a variety of conditions.

Meanwhile, curcumin (Cur), a natural polyphenolic compound derived from *Curcuma longa*, has demonstrated potent antiseptic, anti-inflammatory, and anticancer properties, particularly in MM [8]. Its clinical application is, however, hindered by poor bioavailability and rapid degradation. These concerns can therefore be overcome through the surface-functionalization of FeONPs using Cur, allowing for enhanced bioactive delivery and stability.

IFNα is an additional immunotherapeutic agent for MM, with intrinsic chemotherapy, antiproliferative, and antiviral properties [9,10]. Anticancer combination therapies of IFNα, surgical resection, and chemotherapy has been shown to improve tumor cell elimination and overcome the limitations of monotherapy [11]. However, IFNα is also limited by its systemic toxicity, high cost, and side-effects profile, which leads to poor patient adherence to melanoma treatment [12].

**Figure 1:**
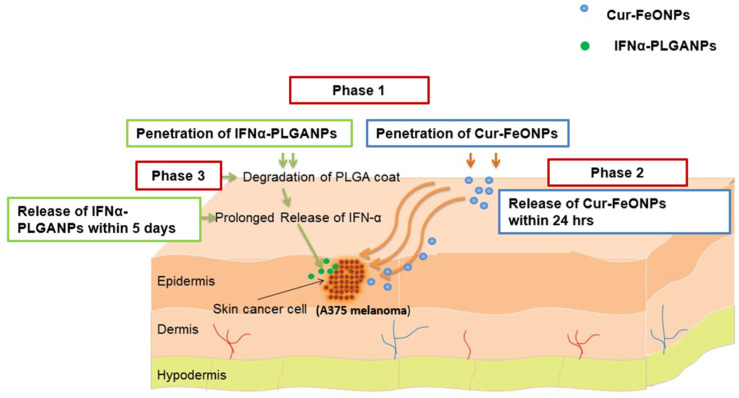
A schematic representation of a potential application of the co-nanoparticulate system for the delivery of Cur and IFNα, where Phase 1 represents penetration of the NPs through the skin, with Phases 2 and 3 depicting the delivery of Cur and IFNα, respectively.

To overcome these limitations, this study has developed and evaluated a dual-release co-nanoparticulate system incorporating fast-release Cur-FeONPs (within 24 h) with prolonged-release IFNα (release over 5 days). This approach has been utilized to leverage the anticancer properties of the individual bioactives, thereby promoting synergistic activity, while minimizing toxicity through a dual-release mechanism using a botanical active (Figure 1 provides a graphical representation of the delivery of Cur and IFNα through the co-nanoparticulate system).

Studies performed include synthesis validation analyses, zeta size and zeta potential evaluations, thermal transition assessments, in vitro drug release studies, and cytocompatibility and antiproliferation analyses using 2D monolayer cultures. These cell viability assessments allow for a practical and informative initial model for assessing cancer cell viability, thereby identifying potential synergistic interactions in response to the varying release profiles assessed in this study. It further allows for an assessment of cytocompatibility against non-cancerous cells to determine the potential of the developed system to selectively target malignant cells. Possible advancements for this analysis include the use of 3D cell models, which can further assess cell–cell and extracellular matrix interactions, while allowing for a more accurate depiction of in vivo performance in the context of bioactive penetration and potential treatment resistance [13,14,15,16].

## 2. Materials and Methods

### 2.1. Materials

Iron (III) chloride hexahydrate (FeCl_3_·6H_2_O), iron (II) chloride (FeCl_2_·4H_2_O), and dimethyl sulfoxide (DMSO) were purchased from Thermo Fisher Scientific (Johannesburg, South Africa), ammonium hydroxide (NH_4_OH) from EMD Chemicals (Modderfontein, South Africa), Cur (95%) from Chem-Impex Int’l Inc. (Wood Dale, IL, USA), and IFNα-2b, polyethylene glycol (PEG), poly(lactic-co-glycolic) acid (PLGA; 50:50), and polyvinyl alcohol (PVA) from Merck Life Science (Pty) Ltd. (Modderfontein, South Africa). NIH-3T3 mouse fibroblast cells (ATCC CRL-1658) were obtained from ATCC (American Type Culture Collection, Manassas, VA, USA), the model malignant melanoma cell line (A375) from Cellonex (Johannesburg, South Africa), the MTT Cell Proliferation Kit I from Roche (Basel, Switzerland), Dulbecco’s Modified Eagle Medium (DMEM) from Life Technologies Limited “Gibco” (Paisley, UK) and Fetal bovine serum from PAN Biotech (Aidenbach, Germany). All other chemicals were utilized as received without further modification.

### 2.2. Synthesis of the Co-Nanoparticle Systems

#### 2.2.1. Cur-FeONPs

The Cur-FeONPs were synthesized using the method described by Bhandari et al., with modifications [7]. Briefly, in a specific setup, 2.2 g of FeCl_3_·6H_2_O and 0.8 g of FeCl_2_·4H_2_O were dissolved in 40.0 mL of water in a three-neck round-bottom flask, with the reaction conducted under a continuous nitrogen flow. As the reaction progressed, 10.0 wt% of Cur (dissolved in DMSO) was gradually added dropwise to the reaction system, at approximately 40 °C. The reaction temperature was then adjusted to 85 °C, with 5.0 mL of 28% (*w*/*w*) ammonium hydroxide introduced into the reaction mixture while maintaining vigorous stirring. The reaction was thereafter allowed to continue stirring at 85 °C for approximately one hour, followed by magnetic decantation and a series of washing steps employing ethanol, a 50:50 mixture of acetonitrile/dichloromethane, and water to isolate the Cur-FeONPs. The Cur-FeONPs were thereafter lyophilized prior to further analysis. As a comparison, FeONPs were prepared without the addition of Cur, as described above.

#### 2.2.2. IFNα-PLGANPs

The IFNα-PLGANPs were prepared using a double emulsion solvent evaporation method. In brief, PEG and PLGA were dissolved in 2 mL of dichloromethane (DCM), which was thereafter mixed with a protein solution consisting of IFNα diluted 20-fold with PBS. The mixture was then emulsified for 30 s using a probe sonicator (Sonics Vibra Cell, Newtown, CT, USA) at 70 W power and a frequency of 20 kHz. This primary emulsion was then added to 3.5 mL of 2 wt% aqueous PVA solution and sonicated for 60 s to form a water/oil/water (w/o/w) double emulsion. The DCM was thereafter evaporated by continuous stirring at 800 rpm for 3 h using a magnetic stirrer (IKA RET B). After complete evaporation of DCM, the solid NPs (IFNα-PLGANPs) were collected, lyophilized, and stored at −20 °C.

### 2.3. Characterization of the Synthesized Nanosystems

#### 2.3.1. Chemical Structure Validation

The analysis of the chemical structure of the synthesized Cur-FeONPs and IFNα-PLGANPs were conducted to detect the distinctive functional groups of Cur, FeONPs (Cur-free NPs), and Cur-FeONPs, as well as on IFNα, PLGA, and IFNα-PLGANPs using a Fourier transform infrared (FTIR) spectrophotometer (Spectrum 100, PerkinElmer Inc, Waltham, MA, USA) and therefore determine the successful synthesis of the respective NPs, while ensuring that no degradation of the bioactives occurred. For each analysis, the dried powder samples were positioned on a diamond ATR crystal, with the spectrum captured within the range of 650 to 4000 cm^−1^ [17].

#### 2.3.2. Particle Size, Zeta Potential, and Surface Morphology

Dynamic light scattering (DLS) and phase-analysis light scattering measurements were conducted on a Zeta Sizer instrument (NanoZS, Malvern Panalytical, Malvern, UK) to determine the average hydrodynamic diameter and zeta potential of the synthesized Cur-FeONPs, FeONPs, and IFNα-PLGANPs. Prior to the analysis, the respective dried samples (10 mg) were suspended in 10 mL distilled water, ultrasonicated, and filtered through a 0.22 µm filter [18]. The samples were then transferred into disposable cuvettes for the hydrodynamic size and polydispersity index (PDI) analysis, followed by zeta potential cuvettes for the surface charge analysis. All analyses were undertaken at a controlled system temperature of 25 °C.

Samples of the Cur-FeONPs, FeONPs, and IFNα-PLGANPs, as obtained, were further subjected to Scanning Electron Microscopy (SEM, ZEISS Electron Microscopy, Carl Zeiss Microscopy Ltd., Cambridge, UK) to assess NP shape and overall morphology. For the SEM analysis, an aliquot of the respective sample was meticulously placed on an aluminum specimen stub before undergoing gold-palladium (AuPd) coating (applied twice). The sample was then observed and imaged under magnifications ranging from 20 to 150 KX.

#### 2.3.3. Determination of Nanoparticle Thermal Phase Stability

Differential Scanning Calorimetry (DSC) was performed on Cur and the synthesized FeONPs and Cur-FeONPs as well as on PLGA, IFNα, and the synthesized IFNα-PLGANPs, using a DSC822E Differential Scanning Calorimeter (PerkinElmer, Waltham, MA, USA) to investigate the thermal properties of the various formulations and bioactives. Prior to analysis, the samples were precisely weighed and placed in a crucible, with an empty crucible serving as the reference. All samples were thereafter subjected to a temperature ramp from 10 to 500 °C at a heating rate of 10 °C/min under a dynamic air atmosphere of 50 mL/min.

#### 2.3.4. Crystallographic Analysis of the Nanosystem Archetypes

X-ray diffractometry (XRD) analysis was conducted on Cur and the synthesized FeONPs and Cur-FeONPs, as well as on PLGA, IFNα, and the synthesized IFNα-PLGANPs using a RIGAKU MiniFlex instrument (RIGAKU Inc., Tokyo, Japan) to evaluate the crystallinity of Cur and IFN-α before and after the respective synthesis processes. Prior to the XRD study, all samples were mounted on aluminum sample holders and analyzed to obtain diffractograms. The following measurement conditions were set for the analysis: a 2θ range of 10–90° and a scan rate at 10 deg/min, with a current and voltage of 15 mA and 40 kV, respectively.

#### 2.3.5. Determination of the Bioactive Loading Within the Nanosystems

The bioactive loading of the Cur-FeONPs was determined by analysis of the supernatant after magnetic decantation using a Thermo Evolution 600 double-beam UV-VIS spectrophotometer (Thermo Fisher Scientific, Waltham, MA, USA). The amount of unloaded Cur in the supernatant was quantified at a fixed wavelength (λ = 428 nm) using a standard quartz cuvette with a 1 cm optical path (ε = 0.0031; R^2^ = 0.996). All experiments were conducted in triplicate, with the bioactive load calculated as the mass of the detected bioactive relative to the total mass of the sample, reported as mean values with standard deviation (SD). The bioactive loading efficiency was determined using the Equation (1):

(1) $$Bioactive\;loading\;efficiency\left(\%\right)=\frac{Total\;amount\;of\;bioactive\;-\;Free\;amount\;of\;bioactive}{Total\;amount\;of\;bioactive}$$

For the bioactive loading analysis of the IFNα-PLGANPs, 10 mg of samples underwent hydrolysis with 1N sodium hydroxide solution (2 mL) under moderate stirring for up to 1 h at room temperature (25 °C) [19]. The resulting solution was thereafter neutralized with 1N hydrochloric acid solution, with the concentration of IFNα determined using the bicinchoninic acid method and a microBCA protein assay reagent kit (Pierce, Rockford, IL, USA) analyzed using a multimodal microplate reader (Victor X3, PerkinElmer, Waltham, MA, USA) at 595 nm in triplicate. The amount of IFNα was thereafter calculated using a standard curve (ε = 0.0022; R^2^ = 0.9972). The encapsulation efficiency (EE) of IFNα was calculated as the ratio of experimental to theoretical IFNα loading, expressed as a percentage. As a control, for all studies quantifying IFNα, a Human IFNα ELISA Kit (Thermo Fisher Scientific, Vienna, Austria) was used to confirm the stability of the loaded IFNα during formulation and throughout the analysis procedure.

The adsorption efficiency (%AE) and Bioactive Loading Capacity (%DLC) were determined by quantifying the free IFNα in the supernatant after the loading procedure. %AE and %DLC were calculated using Equations (2) and (3), respectively.

(2) $$\%AE=\frac{Dl-Ds}{Dl}\times 100$$

where Dl is the initial bioactive amount (ng), and Ds is the free bioactive amount in the supernatant (ng).

(3) $$\%DLC=\frac{Amount\;of\;bioactive\;loaded}{Wf}\times 100$$

where Wf is the total weight of formulation in ng.

#### 2.3.6. In Vitro Release of Bioactives from the Nanosystems

The release profile of Cur from Cur-FeONPs and IFNα from IFNα-PLGANPs was investigated using a protocol adapted from Chircov et al. [20]. For each experiment, 10 mg of each NP system was placed in a cellulose dialysis membrane and incubated in 2.5 mL of PBS solution (pH 7.2) at 37 °C using an orbital shaking incubator rotating at 50 rpm (YIHDER LM-530, YIHDER Co., Ltd., Taipei, Taiwan). Samples (1000 µL) were thereafter collected at predetermined intervals over a 24-h period for Cur-FeONPs and 5 days for IFNα-PLGANPs (4, 8 and 12 h, and 1, 2, 3, 4, and 5 days), centrifuged for 5 min at 5000 rpm using an Eppendorf Centrifuge 5804 (Hamburg, Germany) at room temperature (25 °C) to remove unreacted materials, and analyzed as previously described. To maintain sink conditions, all release media extracted was replenished with an equal amount of fresh release medium.

### 2.4. Nanosystem Cytocompatibility and Antiproliferation Studies

**Figure 2:**
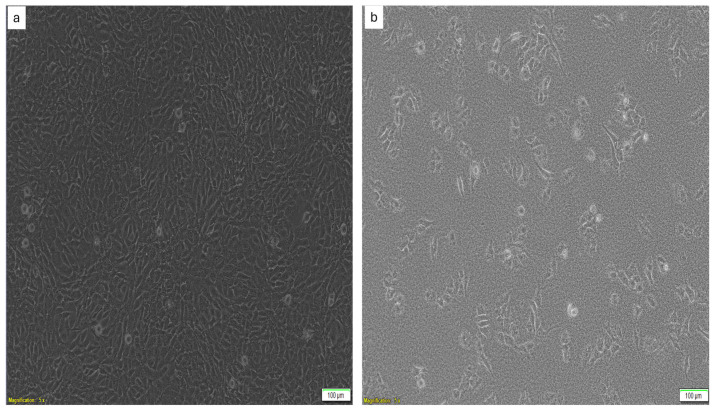
Images of (**a**) the NIH-3T3 cells and (**b**) the A375 melanoma cells used in the cytocompatibility and antiproliferation studies, respectively.

Cytocompatibility and antiproliferation studies were conducted to evaluate the synergistic effects of the synthesized Cur-FeONPs and IFNα-PLGANPs for potential MM skin cancer treatment, using the MTT assay, as described in previous studies [21]. Briefly, for the cytocompatibility analysis, Cur-FeONPs, free Cur, FeONPs, IFNα, and IFNα-PLGANPs and the combination of Cur-FeONPs and IFNα-PLGANPs (1:1) were evaluated on NIH-3T3 cells, as depicted in Figure 2a. For this analysis, the cells were cultured in DMEM supplemented with 10% Fetal Bovine Serum and 1% penicillin/streptomycin and seeded at a density of 5 × 10^5^ cells per well in a 96-well plate. Once confluency was achieved (90%), the cells were treated with concentrations of 10, 5, 2.5, and 1.25 µg/µL of the respective formulations/bioactives. After 72 h of treatment, the supernatants were removed and 10 µL of MTT solution (5 mg/mL) was added to each well, prior to incubation for 4 h at 37 °C under humid conditions with 5% CO_2_. Following this, 100 µL of solubilizing agent (acid-isopropanol; 0.04 N HCl in isopropanol) was added to dissolve the formazan crystals with the plates, which were subsequently incubated overnight at 37 °C with 5% CO_2_. Wells containing only solubilizing agent and growth medium served as blanks, with untreated cells and cells treated with DMSO serving as the positive and negative controls, respectively. The absorbance of the resulting solution was measured in triplicate at 570 nm, with a reference wavelength of 620 nm, using a multimodal microplate reader with cell viability percentages calculated using Equation (4):

(4) $$Cell\;Viability\;\%=\frac{AT}{AC}\times 100$$

where *AT* and *AC* are the absorbance of the treated and untreated cells, respectively.

The antiproliferative effects of the Cur-FeONPs, free Cur, FeONPs, IFNα, and IFNα-PLGANPs, and the combination of Cur-FeONPs and IFNα-PLGANPs (1:1) were analyzed using A375 melanoma cells, as depicted in Figure 2b. These cells were treated with the same concentrations and under identical conditions, as described for the cytocompatibility study for 72 h.

#### Statistical Analysis

Statistical analysis of the cytocompatibility and antiproliferation study results was conducted using SPSS software (version 29, IBM, SPSS Inc., Chicago, IL, USA), with the statistical significance between two experimental groups determined by employing two-sample *t*-tests, while one-way analysis of variance (ANOVA) was performed to assess differences among the various treatment groups and concentrations. Post hoc comparisons were made using the Tukey HSD multiple range tests, with a significance level set at *p* < 0.05. Synergy analysis of the antiproliferation study data was further undertaken using the Bliss Independence model [22].

## 3. Results and Discussion

### 3.1. In Vitro Characterization of the Cur-FeONPs and IFNα-PLGANPs

#### 3.1.1. Assessment of Chemical Stability and Functional Transformation

**Figure 3:**
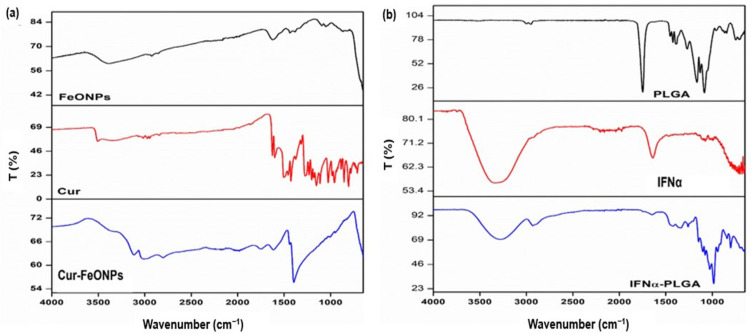
FTIR spectra for (**a**) Cur-FeONPs, free Cur, and the uncoated FeONPs and (**b**) the IFNα-PLGANPs, pure IFNα, and PLGA.

FTIR spectroscopy served as a critical tool for elucidating the functionalization and molecular interactions of the synthesized Cur-FeONPs and IFNα-PLGANPs, as depicted in Figure 3, providing insights into the characteristic functional groups, structural transformations, and chemical stability of the nanoparticulate systems. In the evaluation of the Cur-FeONPs (Figure 3a), the FTIR spectrum of the uncoated FeONPs firstly confirmed the successful synthesis of iron oxide NPs, as evidenced by a prominent absorption peak at 541 and 581 cm^−1^, corresponding to the Fe-O bond [23]. Upon coating with Cur, significant spectral modifications were observed, indicating successful functionalization. This was noted specifically in the disappearance of the peak at 963 cm^−1^, associated with the in-plane bending of the hydroxyl group in the enolic moiety of free Cur, suggesting that functionalization occurred via the keto-enol functionality. Additional spectral features of the Cur-FeONPs further supported the functionalization process. The appearance of a broad peak around 3290 cm^−1^ and a sharp peak around 3470 cm^−1^, corresponding to –OH groups, alongside a shifted and reduced-intensity peak at 1573 cm^−1^ (originally 1605 cm^−1^ in free Cur), indicated interactions between the C=C and C=O groups of Cur and the NP surface [7,24]. Furthermore, the retention of the C-O-C stretching peak at 1023 cm^−1^, albeit with lower intensity, highlights the preservation of aromatic structures during the synthesis process. These results collectively confirm that Cur not only successfully functionalized the NP surface but also acted as a stabilizing agent for the FeONPs.

Evaluation of the IFNα-PLGANPs FTIR spectra similarly provided detailed insights into the molecular interactions of this NP system (Figure 3b). The spectrum of pure PLGA exhibited characteristic peaks, including at 1080 cm^−1^, indicative of C-O-C stretching, 1750 cm^−1^ for C=O stretching, and 2940–3000 cm^−1^ for C-H vibrations of the methyl groups. These findings are consistent with previously reported spectra for PLGA [25]. However, upon encapsulation of IFNα, notable variations were observed, including shifts in the carbonyl and C-O-C stretching peaks to lower wavenumbers. In the single bond region (4000–2500 cm^−1^), the wavenumber range for CH_3_ vibrations in PLGA and IFNα was between 2940 and 3000 cm^−1^. Specifically, IFNα displayed bands at 2920 cm^−1^ (asymmetric CH stretching) and 2870 cm^−1^ (symmetric CH stretching), while in the double bond region (2000–1500 cm^−1^), PLGA was characterized by a representative band at 1750 cm^−1^ and IFNα exhibited bands in the range of 2950–3507 cm^−1^. These findings provided insights into the molecular interactions and bond characteristics of the IFNα-PLGANPs system and proved successful synthesis of the platform [10].

#### 3.1.2. Colloidal Stability and Morphological Analysis

##### Zeta Potential and Particle Size Analysis

**Figure 4:**
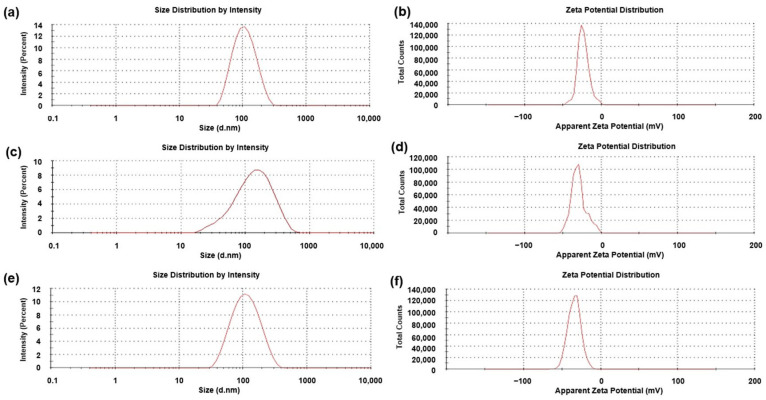
The zeta size and potential profiles of (**a**,**b**) the synthesized FeONPs, (**c**,**d**) the Cur-FeONPs and (**e**,**f**) the IFNα-PLGANPs, respectively.

DLS and zeta potential analyses were performed on the FeONPs prior to and after functionalization with Cur to ascertain the particle size and stability of the system due to the surface modification (Figure 4). The results of these studies indicated a general trend of increase in hydrodynamic diameter and decreased zeta potential values with the addition of Cur. This suggests that the Cur-loading significantly reduced the surface charge of the bioactive delivery system, thereby diminishing their interactions with the surrounding solvent (distilled H_2_O) [20]. Evaluation of the average hydrodynamic diameter of the FeONPs prior to Cur-loading noted a zeta average of 95.37 nm (PDI = 0.151), increasing to 111.00 nm (PDI = 0.279) for the Cur-FeONPs (Figure 4a and Figure 4c, respectively), while the mean zeta potential of the FeONPs was determined to be −23.5 ± 7.43 mV, decreasing to −29.6 ± 9.51 mV for the Cur-FeONPs (Figure 4b and Figure 4d, respectively). This high negative value indicated the non-aggregate nature of the prepared Cur-FeONPs, which is indicative of colloidal stability [21].

These characteristics have the potential to enhance cellular uptake of Cur-FeONPs by human endothelial cells [26]. Furthermore, the nanoparticle size achieved is preferable for the penetration of Cur-FeONPs through the cancer cell membrane, aiding in the accumulation of Cur within cancer cells [27]. The findings by Larese et al. [28] indicated a key distinction between metal and non-metal NPs, emphasizing the impact of particle size after interacting with physiological media, further influencing size-dependent skin penetration. Specifically, NPs smaller than 100 nm are generally considered to be in the optimal size range for deep skin penetration; however this may expose healthy tissue not affected by the skin cancer to potentially toxic bioactives, leading to unwanted adverse effects [28]. Based on this size range, the average size of single Cur-FeONPs developed in this study (≈100 nm) has the potential to localize at the skin cancer site, targeting affected cells, while minimizing diffusion into healthy tissue and systemic circulation.

Evaluation of the mean diameter size and zeta potential of the IFNα-PLGANPs revealed a zeta average of 97.03 nm (PDI = 0.182), with a zeta potential of −34.1 ± 8.32 mV, as presented in Figure 4e and Figure 4f, respectively. The results from the DLS and zeta potential analysis highlighted key physicochemical properties of the IFNα-PLGANPs, critical for their stability and potential therapeutic efficiency, including a hydrodynamic diameter of preferable size for enhanced tumor penetration, with a zeta potential indicative of good colloidal stability.

##### Nanosystem Surface Morphology

**Figure 5:**
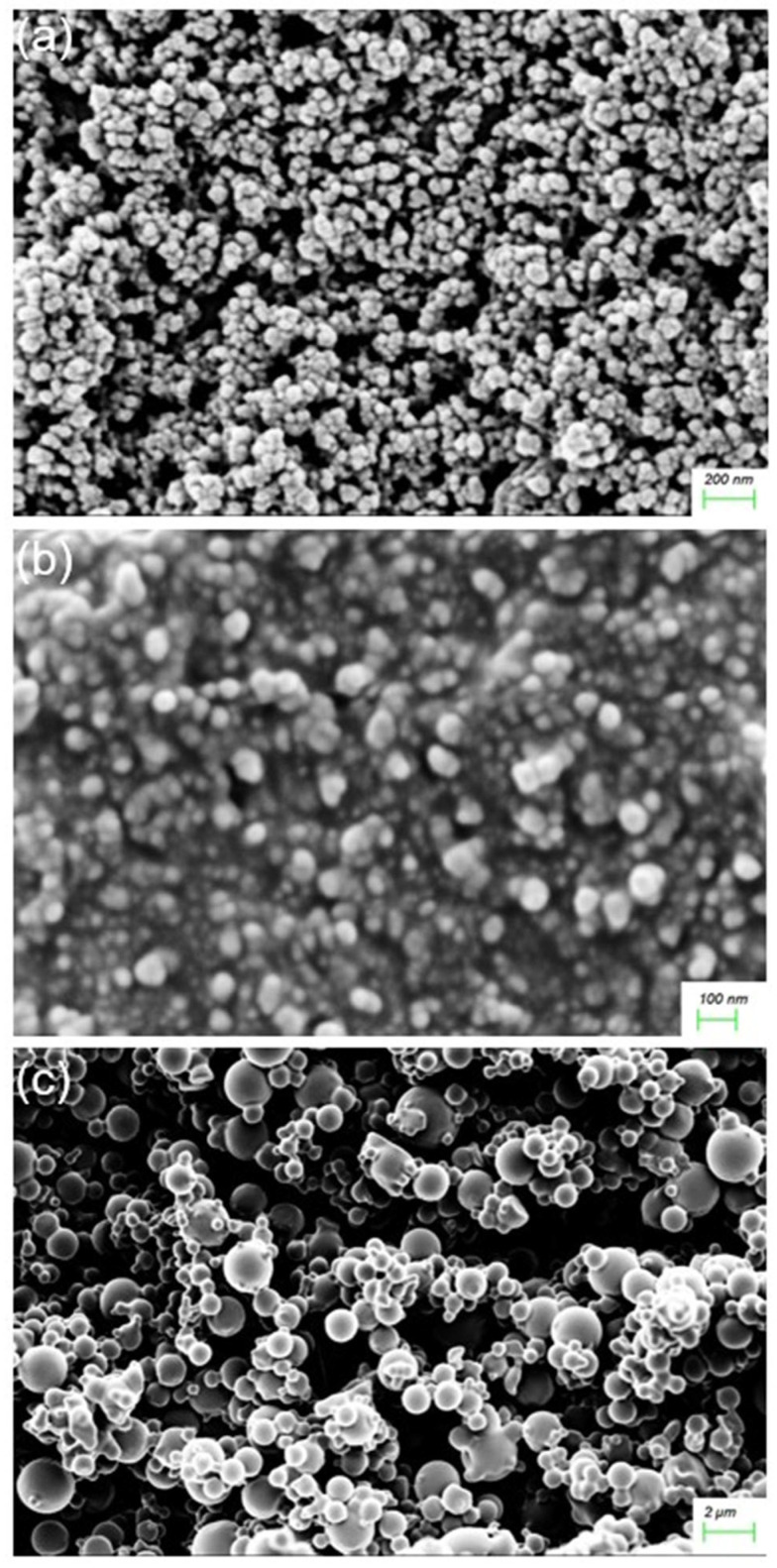
SEM images of (**a**) FeONPs, (**b**) Cur-FeONPs and (**c**) IFNα-PLGANPs.

Among the various methods reported for synthesizing NPs, co-precipitation was chosen as the most suitable method for the preparation of the FeONPs. This method is conducted in an inert atmosphere with alkalinity and elevated temperatures to prevent oxidation, ensuring control over particle size, distribution, and morphology [29]. The synthesis of the FeONPs in this study successfully produced a black-colored powder, characteristic of free FeONPs. After functionalization of the FeONPs with Cur, SEM imagery confirmed that the Cur-FeONPs were spherical with a relatively rugged surface (SEM images before and after functionalization provided in Figure 5a and Figure 5b, respectively). These findings align with previous research indicating that co-precipitation produces spherical NPs, which are advantageous in enhancing bio-distribution and cellular uptake [29]. Representative SEM images of the IFNα-PLGANPs (Figure 5c) similarly revealed that most of the NPs were spherical in shape with a smooth surface. The SEM images noted particles of varying sizes when compared to the DLS data, which can be attributed to the use of as-obtained samples after lyophilization, as well as filtering of the solution prior to DLS measurements. This analysis, however, provided valuable information regarding the shape and surface morphology of the synthesized NPs, which are crucial for their potential bioactivity.

### 3.2. Thermal Phase Analysis

**Figure 6:**
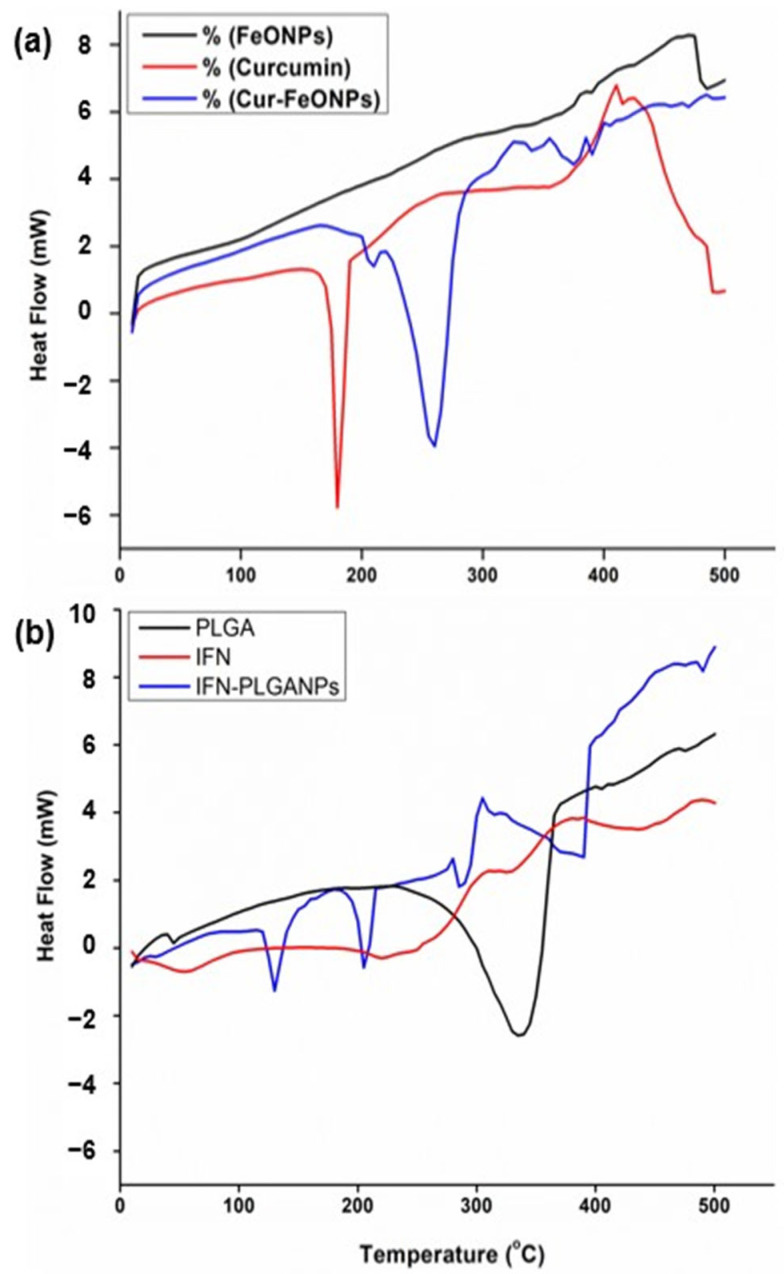
DSC curves of (**a**) FeONPs, free Cur, and Cur-FeONPs and (**b**) PLGA, IFNα and IFNα-PLGANPs, where a negative heat flow is indicative of an exothermic process, and a positive heat flow is indicative of an endothermic process.

Figure 6a illustrates the DSC curves of free Cur, FeONPs and Cur-FeONPs, with the results of the DSC analysis noting that the unfused Cur displayed a distinct endothermic peak with a melting point at 186.2 °C [30], whereas the melting point of Cur-FeONPs ranged between 285 and 300 °C. Notably, the melting point of the FeONPs exceeded the specified range due to the physical properties of iron, and the Cur-functionalized FeONPs exhibited a forward shift with a considerable decrease in peak intensity. This shift could be attributed to the interactions between Cur and iron oxide, which increased the stability of the system. The exothermic peak observed in the DSC analysis of FeONPs at approximately 480 °C corresponds to the phase transition from maghemite to hematite, a transformation typically occurring around 500 °C [31,32].

Similarly, the DSC analysis confirmed the chemical interaction between PLGA and IFNα in the synthesis of the IFNα-PLGANPs (Figure 6b). Evaluation of the thermogram of free PLGA noted that the sample does not exhibit a distinct melting point, confirming its amorphous nature. The free PLGA polymer further displayed a small peak at 50 °C, corresponding to the relaxation peak following glass transition, with another peak at 340 °C attributed to the thermal decomposition of the polymer. The DSC curves indicated that free PLGA is thermally stable up to 250 °C, aligning with the study by Mukerjee and Vishwanatha [33]. Additionally, analysis of the pure IFNα DSC profile revealed an exothermic peak at approximately 60 °C, aligning with the denaturation of the molecule. Evaluation of the IFNα-PLGANPs further noted that the preparation method did not affect the PLGA polymer structure. The thermogram displayed peaks at approximately 130 °C, 200 °C, and 290 °C, indicating that IFNα is entrapped in the PLGA matrix. This suggests that the bioactive is incorporated into the NPs and is in an amorphous or disordered crystalline phase, forming a molecular dispersion within the polymer matrix.

### 3.3. Polymorphic Evaluation of the Nanosystems

**Figure 7:**
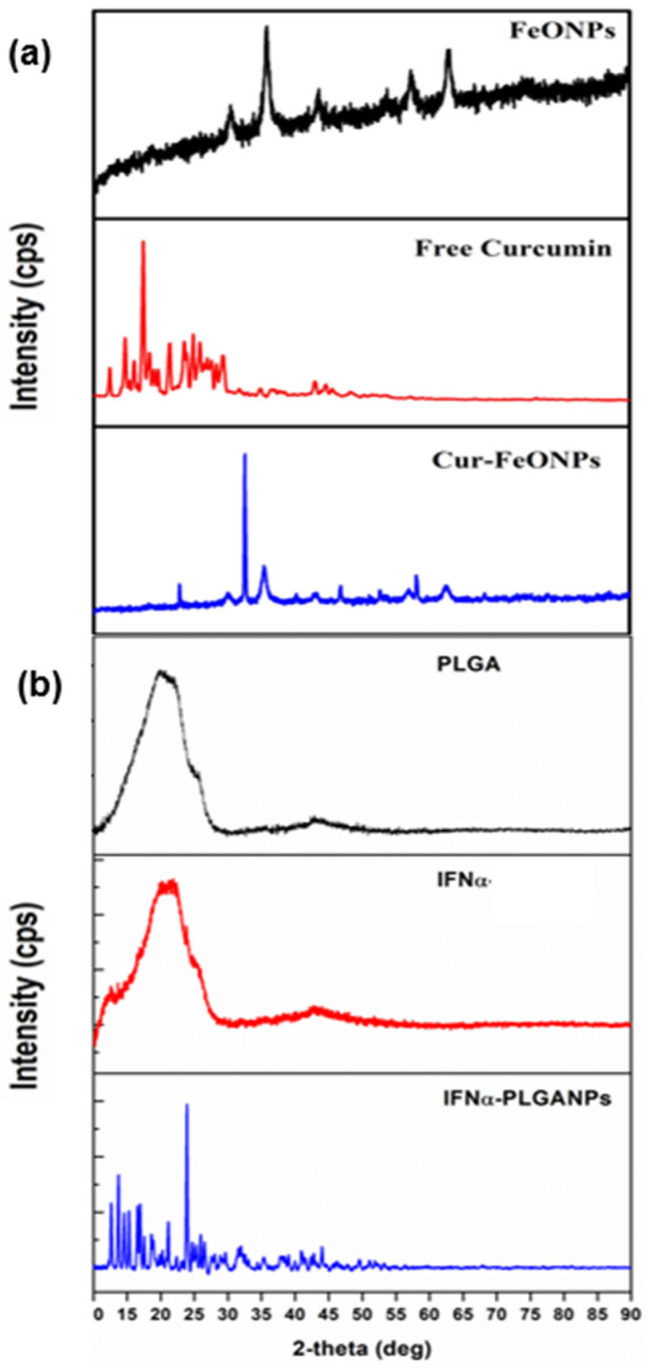
Images of the XRD spectra for (**a**) FeONPs, free Cur, and Cur-FeONPs and (**b**) PLGA, pure IFNα, and IFNα-PLGANPs.

The XRD pattern of Cur-FeONPs displayed characteristic peaks at 2θ = 30.2, 35.5, 43.2, 53.6, 57.0, and 62.8. From this, it is evident that the diffraction peaks of Cur-FeONPs are closely aligned with the typical diffraction peaks of Fe_3_O_4_, as shown in Figure 7a. The XRD profile for Cur-FeONPs further pointed towards the formation of nanocrystals, present as self-consistent units on the NP surface, with previous research noting that Cur and similar materials can crystallize on these surfaces, potentially leading to novel structural and functional properties [34]. The variation in peak intensity between FeONPs and Cur- FeONPs could also be attributed to the Cur coating process, which resulted in a reduction in peak intensity, as both samples displayed high-intensity peaks within the 10–30° range of the 2θ diffraction angle. This indicated the crystalline nature of Cur in its pure form and around the NPs, which is consistent with prior studies, confirming that the Cur molecule remained intact without degradation during NP synthesis, preserving its structural integrity in the formulation [35,36].

The XRD patterns of IFNα-PLGANPs additionally revealed numerous sharp peaks indicative of crystalline phases (Figure 7b). Specifically, the XRD data, recorded in the 2θ range of 10–90°, showed high-intensity peaks at 2θ angle values of 14.6°, 15.4°, 17.5°, 21.9°, 21.1°, and 23.9° for the particle system. These peaks closely resemble those reported by Singh et al. [37]. The presence of sharp, intense peaks suggests the presence of the crystalline form of the bioactive. In contrast, the XRD spectrum of free PLGA displayed no peaks but a broad amorphous band between 10° and 90°, indicating the amorphous nature of PLGA as a copolymer, with a similar profile generated for free IFNα.

### 3.4. Bioactive-Loading Capacity and Adsorption Efficiency

The loading capacity and adsorption efficiency of the Cur-FeONPs and IFNα-PLGANPs were evaluated to elucidate the efficiency of bioactive incorporation into the NPs. For the Cur-FeONPs, the Cur-loading efficiency was found to be approximately 93.83% ± 0.1, indicating a high loading capacity and entrapment efficiency (%EE). This result is consistent with findings by Chircov et al., where the loading efficiency was largely influenced by the surface area of the metal nanocarriers [20]. The high %EE suggests that the Cur used during synthesis successfully interacted with the NP platform, reflecting optimal conditions for Cur incorporation. In contrast, the protein loading efficiency for IFNα was 78.9% ± 0.3, which can be attributed to the experimental conditions used in the synthesis process, including the volume and concentration of protein in the inner aqueous phase, the polymer concentration in the organic phase, the type of polymer used, the emulsification times, and the additives included at different phases of NP assembly [38].

### 3.5. In Vitro Cur and IFNα Release in Biorelevant Media

**Figure 8:**
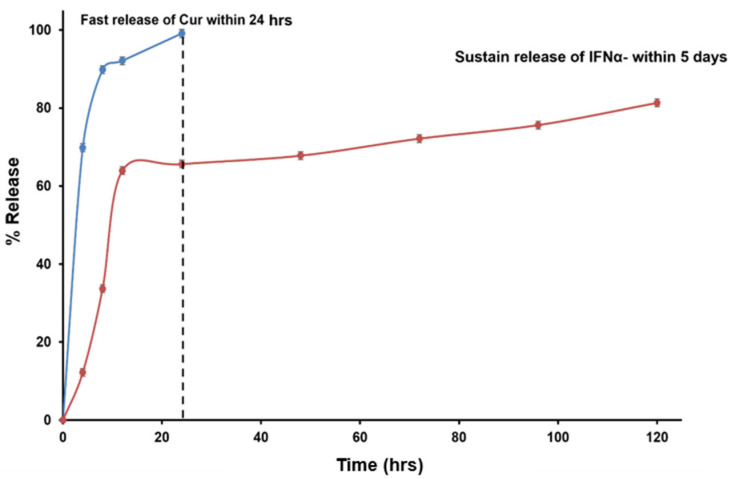
Bioactive release profiles of Cur from the Cur-FeONPs and IFN-α from the IFNα-PLGANPs.

The release profiles of Cur-FeONPs and IFNα-PLGANPs provided critical insights into their bioactive delivery dynamics and potential biomedical applications (Figure 8). The release profile of Cur from Cur-FeONPs aligns with the findings from Chircov et al., who reported that nanocarriers exposed to prolonged reaction times exhibited slightly faster release rates due to their larger size. However, in this study, nanocarrier synthesized demonstrated higher loading efficiency, resulting in an enhanced overall release of Cur. Sustained release was observed between 8 and 24 h, highlighting the system’s potential for extended bioactive delivery applications, when needed [20].

The release of IFN-α from IFNα-PLGANPs indicated that the release corresponded to a slower, sustained phase, likely due to the induction period before the onset of PLGA polymer bioerosion, where the hydrolysis of ester bonds in PLGA led to the formation of channels through which IFN-α could diffuse. However, the incomplete release of IFNα (cumulative release of 81.3% after 5 days) was attributed to the high-molecular-mass polymer fragments that restricted the diffusion of the water-soluble cytokine. These fragments likely shielded IFNα from exposure to the outer liquid phase, slowing its release through the porous NP matrix. Interestingly, the findings from Feczkó et al. offered a contrast to this study, where IFN-α release from PLGA-based NPs demonstrated remarkable sustained release properties, with 90% release achieved after the 16-day release study. Notably, pegylated IFNα encapsulated in pegylated-PLGA (PEG-IFN-PEG-PLGA) exhibited a sustained release profile, with encapsulated bioactive levels reaching up to 117% of the baseline concentration, while the non-pegylated IFNα-PLGA formulations showed lower release rates, with cumulative levels between 60% and 70% of baseline after 16 days [39].

These findings highlight the distinct release mechanisms and efficiencies of Cur-FeONPs and IFNα-PLGANPs. While Cur-FeONPs demonstrated a rapid and complete release suitable for short-term applications, the IFNα-PLGANPs exhibited a controlled, sustained release profile with potential for prolonged therapeutic effects.

### 3.6. Cytotoxicity Assessment

#### 3.6.1. Cytocompatibility

**Figure 9:**
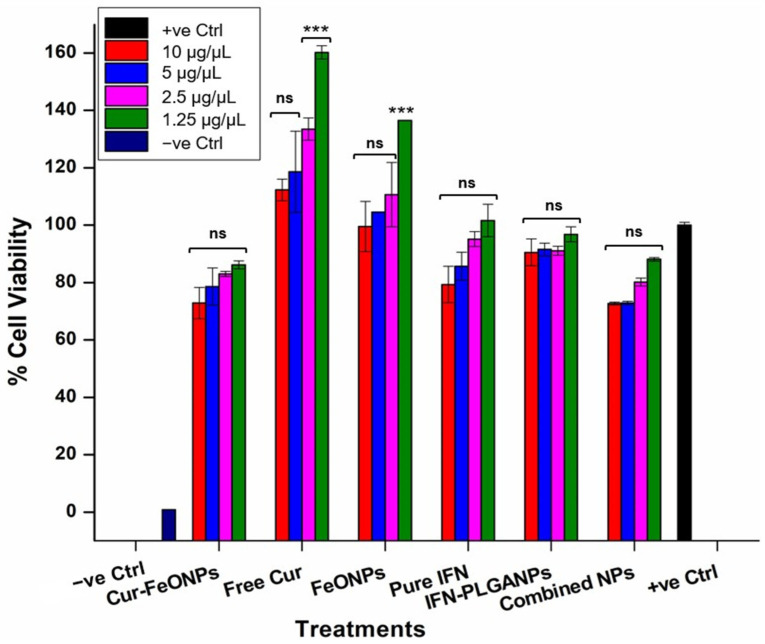
Cytotoxicity assessments to evaluate the cell viability of Cur-FeONPs, free Cur, FeONPs, pure IFNα, IFNα-PLGANPs, and the combination of Cur-FeONPs and IFNα-PLGANPs at different concentrations on NIH-3T3 cells 10, 5, 2.5, and 1.25 μg/μL, respectively. All experiments were undertaken for 72 h (*n* = 3). ns = non-significant; *** = *p* ≤ 0.001.

The potential toxicity of nanoparticulate systems is a key consideration in their biomedical and pharmaceutical applications, requiring careful assessment to ensure that they do not adversely affect healthy cells. In vitro safety studies offer a cost-effective and efficient analysis prior to in vivo animal testing, addressing both logistical and financial challenges [40]. The MTT assay was therefore employed to assess the cytotoxic effects of the Cur-FeONPs, free Cur, FeONPs, pure IFNα, IFNα-PLGANPs, and the combination of Cur-FeONPs and IFNα-PLGANPs (1:1) on NIH-3T3 fibroblast cells, seeded at a density of 5 × 10^5^ cells. Cell viability was measured after 72 h of exposure to varying concentrations of the compounds (Figure 9), corresponding to 10, 5, 2.5, and 1.25 µg/µL, respectively. The results indicated that NIH-3T3 cells maintained a viability above 70% across all tested concentrations, with Cur-FeONPs demonstrating relatively high compatibility, even at the maximum concentration of 10 µg/µL. Importantly, the combined NP treatment also maintained high cell viability across all concentrations, confirming its biocompatibility and safety profile when applied to healthy fibroblast cells under the given experimental conditions. It was noted that for the free Cur and FeONP groups, specifically at the lowest concentrations (2.5, and 1.25 µg/µL), cell viability increased substantially. This increase displayed for the free Cur group is consistent with previous studies assessing the influence of low-concentration curcumin on fibroblast viability, which noted increases above 160% after 24–72 h, aligning with the anti-inflammatory, maturation, and proliferative effects of Cur [41,42,43]. Similarly, previous research has shown that exposure of low concentrations of FeONPs to fibroblast cells is associated with increased cell proliferation between 125 and 140% after 72–120 h, which is attributed to the stimulation of growth factor release and the modulation of the cellular environment [44,45].

Statistical analysis of the cytocompatibility study results indicated non-statistically significant differences in the cell viability between the untreated sample (positive control) and the majority of the treatment formulations (*p* > 0.5), with the highest significant differences being for the lowest concentrations of FeONPs and free Cur (*p* = 0.001). This can be attributed to the substantially higher viabilities attained for these samples and not due to cytotoxicity. These results therefore suggest that the tested formulations did not exhibit cytotoxicity towards the NIH-3T3 cells and maintained strong cell viability compared to the untreated group. Furthermore, no significant differences were observed among the different concentrations of Cur-FeONPs, nor between Cur-FeONPs and FeONPs and free Cur, or between IFN-PLGANPs and pure IFN, suggesting that the formulations maintained consistent, non-cytotoxic activity after their respective bioactive loading.

Interestingly, for the combined NP formulations, no significant differences were detected either when compared to the positive control or between the various concentrations within the sample group itself (*p* > 0.5). Additionally, when the NP-combined formulations were compared with FeONPs, there were no statistically significant differences observed, even at the highest concentration (10 µg/µL, *p* = 0.077). In contrast, however, the NP-combined treatments exhibited statistically significant differences when compared with free Cur (*p* = 0.001), reinforcing the different biological interactions of the NP combination.

#### 3.6.2. Antiproliferation Activity

**Figure 10:**
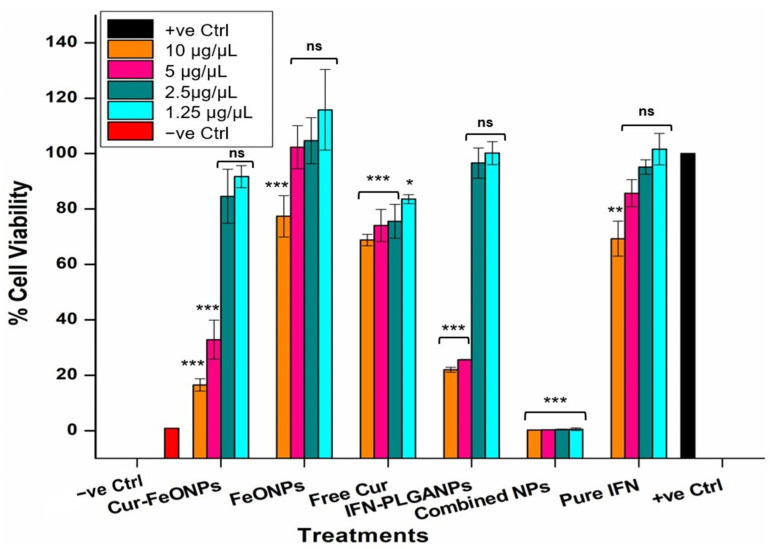
Cytotoxicity of A375 cells under different concentrations for the Cur-FeONPs, IFNα-PLGANPs, the combination of Cur-FeONPs and IFNα-PLGANPs, pure IFN-α after 72 h (*n* = 3). ns = non-significant; * = *p* ≤ 0.05; ** = *p* ≤ 0.01; *** = *p* ≤ 0.001.

The tumor growth inhibition properties of the Cur-FeONPs and IFNα-PLGANPs were evaluated on the A375 melanoma cell line to ascertain their potential anticancer effects alone and in combination (Figure 10). All tested compounds inhibited A375 cell proliferation at the highest concentration analyzed (10 μg/μL) when compared to the positive control, demonstrating selective toxicity to the cancerous cells, with minimal impact on NIH-3T3 cells, as determined in the cytocompatibility study. Specifically, at this concentration, the IFNα-PLGANPs showed no significant difference in suppressing A375 cells, when compared to the corresponding Cur-FeONPs sample (<5% difference in cell viability). This result, however, confirmed that the IFNα-PLGANPs inhibited proliferation in A375 cells, with IC50 values of 0.47 and 0.31 μg/μL, after 72 h of treatment. It was further determined that the sustained release properties of the IFNα-PLGANPs had the greatest influence on A375 cell viability at the two highest concentrations, when compared to pure IFNα. This result confirmed that the IFNα-PLGANPs protected IFNα from degradation and allowed for controlled release, potentially enabling lower doses to be used for anticancer effects. Similarly, the free Cur and Cur-FeONPs displayed concentration-dependent cytotoxicity against the A375 cells, with the greatest effect on A375 cell viability occurring at 10 and 5 μg/μL. It was further noted that the synthesized FeONPs without Cur functionalization had limited activity against the A375 cells at the concentrations 5, 2.5, and 1.25 μg/μL, thereby determining that these NPs functioned purely as a carrier without inducing significant inhibitory effects at these concentrations, with only a limited activity noted at this highest concentration (10 μg/μL; 22% inhibition).

This approach further investigated the combination of IFNα-PLGANPs alongside Cur-FeONPs, focusing on maintaining effective individual doses. This strategy aimed to demonstrate the efficacy of combining controlled-release mechanisms within a single intervention, offering a promising avenue for synergistic anticancer effects. Both nanosystems, when used individually, showed cytotoxic activity against A375 melanoma cells in the highest concentration dose (10 μg/μL) with no effect in the lowest concentration (1.25 μg/μL). However, when combined, the Cur-FeONPs and IFNα-PLGANPs demonstrated a synergistic effect, significantly reducing A375 melanoma cell viability more effectively than either formulation alone, with viabilities of less than 1% achieved across all concentrations (0.283% ± 0.034, 0.318% ± 0.044, 0.415 ± 0.166, and 0.539% ± 0.49, for concentrations 10, 5, 2.5, and 1.25 μg/μL, respectively).

Statistical analysis of these results demonstrated non-statistically significant differences in cell viability between the positive control and all tested concentrations of the individual (non-combined) treatments, except at the highest doses (10 and 5 μg/μL) of Cur-FeONPs (*p* = 0.001) and IFNα-PLGANPs (*p* = 0.049), and the 10 μg/μL samples for FeONPs and pure IFN. Statistical differences were also noted across all free Cur concentrations, with the lowest concentration (1.25 μg/μL) having the lowest significance (*p* = 0.024). These results indicate a notable inhibitory effect of both NP formulations at high concentrations compared to the untreated cells and lower doses, which showed no significant differences (*p* > 0.05). In contrast, the combined NP group did not show statistically significant differences when compared to the high-dose treatments of Cur-FeONPs and IFNα-PLGANPs (*p* = 1.00 and 0.957, respectively), suggesting comparable potency at elevated concentrations. Additionally, within-group analysis of the NP-combined samples revealed no significant variation across their different concentrations (*p* = 0.534), indicating a stabilized dose-response profile. However, inter-group comparisons confirmed that the NP-combined samples were significantly different from all other treatment groups, including the untreated control (*p* < 0.05), supporting the formulation’s distinct and synergistic efficacy.

Synergy analysis of the combined treatments in comparison to the individual Cur-FeONPs and IFNα-PLGANPs using the Bliss Independence model corresponded with this observation (Table 1), with the highest concentrations of the NPs displaying an independent interaction, thereby noting no synergy However, the combination treatments at 5, 2.5, and 1.25 μg/μL displayed significant synergy, with the lowest concentration (1.25 μg/μL) having an excess viability of 91.32%. The combination of the synthesized NPs therefore possesses a synergistic interaction, validating the enhanced antiproliferative activity observed.

This enhanced anticancer efficacy displayed for the combined NPs could therefore be attributed to the complementary mechanisms of the platforms: Cur-FeONPs delivered through a rapid release, while the IFNα-PLGANPs provided sustained anticancer activity. The combined treatment of Cur-FeONPs and IFNα-PLGANPs therefore exhibited selective toxicity against A375 melanoma cells, while preserving the viability of NIH-3T3 fibroblast cells, highlighting its potential specificity and safety.

It should be noted, however, that while 2D cell culture models, such as monolayer cultures, are widely used in preclinical research due to their relevance to vivo cellular behavior in platforms assessing anticancer activity, 3D models may demonstrate superiority due to their ability to replicate complex interactions [46]. This is because, unlike 2D models, which lack structural and physiological complexity, 3D cultures better simulate the tumor microenvironment, preserving cellular heterogeneity, signaling pathways, and exosome activity, making them more suitable for cancer research and drug evaluation. The evaluation of the formulated IFNα-PLGANPs in combination with Cur-FeONPs using 3D cell culture models is therefore a possible next step in determining the efficacy of the co-nanoparticulate system for potential cancer treatment. Additionally, while this study did not evaluate the mechanism of activity of the individual NP platforms and the combination treatment, this may provide a further perspective on the nanosystem as part of future studies exploring the same or different combinations.

## 4. Conclusions

This study presents a novel co-nanoparticulate system for the dual release of Cur and IFNα from functionalized FeONPs and PLGANPs, respectively. The successful synthesis and characterization of both nanosystems (i.e., Cur-FeONPs and IFNα-PLGANPs) highlighted their structural stability, biocompatibility, and enhanced efficacy, with the Cur-FeONPs displaying maximum release after 24 h and the IFNα-PLGANPs achieving sustained IFN-α release over five days. Both platforms additionally demonstrated selective cytotoxicity, showing significantly higher antiproliferative activity against melanoma (A375) cells, while exhibiting minimal toxicity toward normal fibroblast cells (NIH-3T3). This was confirmed through synergy studies, which indicated significant synergy of the combined NPs at the lowest concentrations for activity against A375 cells. Therefore, by integrating bioactives with complimentary mechanisms of action and sustained release, the combined nano-formulations provide a synergistic prototype for enhanced activity in A375 melanoma cells. Potential applications of this combined system include topical administration, as well as intradermal delivery, which can minimize systemic exposure and reduce the likelihood of off-target effects commonly associated with oral or intravenous administration.

With the promising results achieved, further investigation through additional in vitro studies, preclinical evaluation, and subsequent clinical trials can be conducted, including the potential for use of the developed platforms in further combination therapies to overcome chemotherapy resistance. Additionally, while this study primarily focused on the formulation and immediate characterization of the NPs, the assessment of the long-term colloidal stability of the synthesized NPs may also be conducted for translational applications, as well as cytotoxicity evaluation using 3D cell culture. Further studies into the mechanism of activity of the co-nanoparticulate system may also be performed, as well as analysis of the NP platforms in simulated tumor microenvironment conditions to ascertain the potential for pH-responsive release.

## Figures and Tables

**Table 1:** Synergy Analysis of the Combined NP Treatments Using the Bliss Independence Model.

Concentration (µg/µL)	Expected Viability (%)	Excess Viability (%)	Interaction
10	3.63 ± 0.15	−3.35 ± 0.17	Independent
5	8.42 ± 0.21	−8.10 ± 0.24	Synergistic
2.5	81.70 ± 1.75	−81.29 ± 1.79	Synergistic
1.25	91.86 ± 2.10	−91.32 ± 2.15	Synergistic

## Data Availability

The data are available from the authors upon request.

## Abbreviations

The following abbreviations are used in this manuscript:

2D	Two-dimensional
3D	Three-dimensional
Cur	Curcumin
Cur-FeONP	Curcumin-functionalized Iron Oxide Nanoparticle
DCM	Dichloromethane
DMEM	Dulbecco’s Modified Eagle Medium
DMSO	Dimethyl Sulfoxide
FeONP	Iron Oxide Nanoparticle
FTIR	Fourier Transform Infrared
IFNα	Interferon alpha
IFNα-PLGANP	Interferon alpha-loaded Poly(lactic-co-glycolic) acid Nanoparticle
MM	Malignant Melanoma
MTT	3-(4,5-Dimethylthiazole-2-yl)-2,5-diphenyl tetrazolium bromide
NMSC	Non-melanoma Skin Cancer
NP	Nanoparticle
PBS	Phosphate Buffered Saline
PDI	Polydispersity Index
SD	Standard Deviation
SEM	Scanning Electron Microscopy
XRD	X-Ray Diffraction
